# Effect of Rosmarinic and Caffeic Acids on Inflammatory and Nociception Process in Rats

**DOI:** 10.5402/2011/451682

**Published:** 2011-03-30

**Authors:** Giovana Duzzo Gamaro, Edna Suyenaga, Milene Borsoi, Joice Lermen, Patrícia Pereira, Patrícia Ardenghi

**Affiliations:** ^1^Departamento de Bioquímica, Universidade Federal de Pelotas, Campus Capão do Leão S/N Caixa Postal 354, 96010-900 Pelotas, RS, Brazil; ^2^Instituto de Ciências da Saúde, Universidade Feevale, RS 239, 2755, 93352-000 Novo Hamburgo, RS, Brazil; ^3^Instituto de Ciências Básicas da Saúde, Universidade Federal do Rio Grande do Sul, Rua Sarmento Leite, 500, 107, 90046-900 Porto Alegre, RS, Brazil; ^4^Universidade Luterana do Brasil, Curso de Farmácia, Rua Miguel Tostes 101, 92420-280 Canoas, RS, Brazil; ^5^Fundação Estadual de Produção e Pesquisa em Saúde/Centro de Desenvolvimento Científico e Tecnológico, Avenue Ipiranga, 5400, 90610-000 Porto Alegre, RS, Brazil

## Abstract

Rosmarinic acid is commonly found in species of the *Boraginaceae* and the subfamily *Nepetoideae (Lamiaceae)*. It has a number of interesting biological activities, for example, antiviral, antibacterial, anti-inflammatory, and antioxidant. The aim of the present study was to investigate the effect of the i.p. administration of caffeic and rosmarinic acid (5 and 10 mg/kg) on anti-inflammatory and nociceptive response using carrageenan-induced pleurisy model and tail-flick assay in rats. The analysis of cells in the pleural exudates revealed a reduction of 66% of the number of leukocytes that migrated to the pleural cavity in the animals treated with 5 mg/kg caffeic acid, and of 92.9% for the animals treated with 10 mg/kg in comparison with the control group. These exudates showed a balanced distribution of polymorphonuclear (PMN) and mononuclear (MN) cells, differently from the control group, in which PMN cells were predominant. The analysis to tail-flick latency was increased in the group treated with 10 mg/kg caffeic acid characterizing a nociceptive response. While there was no difference between control group and animals treated with rosmarinic.

## 1. Introduction

Inflammation or phlogosis is a pathophysiological response of mammalian tissues to a variety of hostile agents including infectious organisms, toxic chemical substances, physical injury, or tumor growth leading to local accumulation of plasmic fluid and blood cells [1]. 

Although inflammation is a defense mechanism, the complex events and mediators involved in the inflammatory reaction can induce, maintain, and aggravate many disorders. This process is invariably characterized by the production of prostaglandins, leukotrienes, histamine, bradykinin, platelet-activating factor (PAF), and by the release of chemicals from tissues and migrating cells [2, 3]. It is well known that the central feature of inflammatory activity is the activation of phagocytic cells that synthesize and release large amounts of reactive species, causing cell and tissue injury, often directly by oxidative degradation of essential cellular components [4]. 

Following tissue injury and inflammation, nociceptors are sensitized, causing previously mild or ineffective stimulation becomes painful [5]. In acute pain, nociceptive transmission comprises a network of neuronal arrangement distributed between peripheral, spinal, and supraspinal levels. This occurs through the action of neurotransmitters, neuromodulators, and intracellular messengers [6, 7].

Plants were historically the main source for obtaining therapeutic agents. Natural selection and competition among species led to the synthesis of secondary metabolites, and many are used as drugs with marked biological activities [8]. 

Rosmarinic acid is an ester of caffeic acid and 3,4-dihydroxyphenyllactic acid. It is commonly found in Boraginaceae species and in the Nepetoideae subfamily of the Lamiaceae. It is also found in species of other higher plant families, and in some fern and hornwort species [9]. Rosmarinic acid has a number of interesting biological activities, for example, adstringent, antioxidative, antimutagen, antibacterial, and antiviral effects [10–16].

The anti-inflammatory properties of rosmarinic acid are thought to be based on the inhibition of lipoxygenase and cyclooxygenases, and on the interference of rosmarinic acid with the complement cascade [9, 17–19] and the inhibition of expression of inflammatory cytokines [20]. Caffeic acid also inhibits 5-lipoxygenase (5-LOX) and protein kinase C (PKC) activity [21, 22] besides antioxidant activity [23–26].

Therefore, the aim of this study was to investigate and compare the effects of rosmarinic and caffeic acids *in vivo *on inflammatory and nociceptive processes using carrageenan-induced pleurisy model and tail-flick assay in rats.

## 2. Materials and Methods

### 2.1. Animals

Male Wistar rats (12-week-old, weighing 200–300 g) from the animal unit of Universidade Feevale were used in this study. All animals were housed at 25 ± 1°C with 12 hours light, 12 hours dark cycle and supplied with standard rat chow and water freely available. Animals were handled and cared according to NIH Guide for the Care and Use of Laboratory Animals and the Brazilian Society for Neuroscience and Behavior (SBNeC) recommendations for animal care. This study was submitted to Ethical Committee of Feevale and received approval by code no. 2.08.03.07.673.

### 2.2. Drugs and Pharmacological Procedure

Rosmarinic acid and caffeic acid were purchased from Sigma Chemicals (St. Louis, Mo., USA). Thirty minutes prior to procedures, animals were pretreated. For pleurisy model, rats were divided in five groups (*n* = 7 animals/group). Caffeic acid (5 and 10 mg/kg) and rosmarinic acid (5 and 10 mg/kg) were administered intraperitoneally, using saline as vehicle. The control group were received saline (2 mL/kg) by the same route. For tail-flick test animals were divided in three groups (*n* = 10 animals/group) and rats were treated intraperitoneally with 10 mg/kg caffeic acid or rosmarinic acid. The control group animals received the same experimental handling as those of the test groups except that the drug treatment was replaced with appropriate volumes of the dosing saline.

### 2.3. Pleurisy Induction

Rats anaesthetized with ethyl ether, and injected intrapleurally with 0.1 mL of a 1 mg/mL solution of carrageenan, as described by Spector [27]. Four hours later, the animals were sacrificed with ethyl ether and the pleural cavities were exposed. The exudates were collected and the cavity was flushed with 2 mL of phosphated-buffered saline (PBS). The total leukocyte number in the pleural exudate was counted in a Neubauer chamber. Slides of the cellular exudate were also prepared and differential cell counting was performed. In this experiment leukocytes accumulation in the peripheral blood was also determined. Rats were first anaesthetised and blood samples were taken from the tail before and after 4 hours of the carrageenan injection for differential leukocyte counting.

### 2.4. Antinociception Recording

Nociception was assessed with a tail-flick apparatus (Insight EFF-300) [28]. Rats were wrapped in a towel and placed on the apparatus, the light source positioned below the tail was focused on a point 2-3 cm rostral to the tip of the tail. Deflection of the tail activated a photocell and automatically terminated the trial. Normal response latencies were usually between 2.5 and 3.0 seconds, and a 10 seconds cutoff was used to prevent tissue damage. Tail-flick latency represented the period of time from the beginning of the trial to the tail deflection. On day 1, subjects were familiarized with the tail-flick apparatus. This was done because it has been observed that novelty itself can induce antinociception [29]. On day 2, animals were submitted to the tail-flick measurement. 

### 2.5. Statistical Analysis

Data from pleurisy are expressed as mean ± standard error (S.E.M.). Comparisons between counting before and after pleurisy induction were performed using paired Students *t*-test. ANOVA and Tukey test were used to compare the treatment groups. Latency to tail-flick (nonparametric data) are expressed as median (interquartil range) and were analyzed by a Mann-Whitney *U*-test (independent samples) or by Wilcoxon Matched-Pairs Test (related samples).

## 3. Results

### 3.1. Effects of Caffeic and Rosmarinic Acids on Carrageenan-Induced Pleurisy

Total number of leukocytes showed a significant increase in both groups (treated and control) in Table 1. The leukocytes recruited to the peripheral circulation, which is primarily indicated by the increase in the number of neutrophils after the induction of inflammation.

The analysis of cells in the pleural exudates revealed a reduction of 66% of the number of leukocytes that migrated to the pleural cavity in the animals treated with 5 mg/kg caffeic acid, and of 92.9% for the animals treated with 10 mg/kg in comparison with the control group. These exudates showed a balanced distribution of polymorphonuclear (PMN) and mononuclear (MN) cells, as shown in Table 2, differently from the control group, in which PMN cells were predominant.

The administration of 5 and 10 mg/kg produced marked increases in total leukocyte counts after inflammation induction. However, only the animals treated with 10 mg/kg showed a significantly greater number of neutrophils than the animals in the control group, both before and after the administration of carrageenan (Table 1), as well as a significant increase in the number of lymphocytes after pleurisy induction in comparison with the control group. The analysis of cells that migrated to the pleural exudate revealed a significant reduction in leukocyte recruitment in comparison with the control group, with inhibition of 78.2% and 80.7% for animals treated with 5 and 10 mg/kg. No predominance of PMN or MN cells was found in the exudate (Table 2).

The comparison of effects of the treatment with rosmarinic acid or caffeic acid on the inhibition of leukocyte migration to the inflammation sites revealed a significant difference in the group of animals treated with 10 mg/kg caffeic acid in comparison with the other groups. There was a reduction of 79.2% in the number of total exudate cells in the group that received 5 mg/kg caffeic acid, and of 67.6% and 63.3% in the group treated with 5 and 10 mg/kg of rosmarinic acid.

### 3.2. Effects of Rosmarinic and Caffeic Acids on Tail-Flick

**Figure 1:**
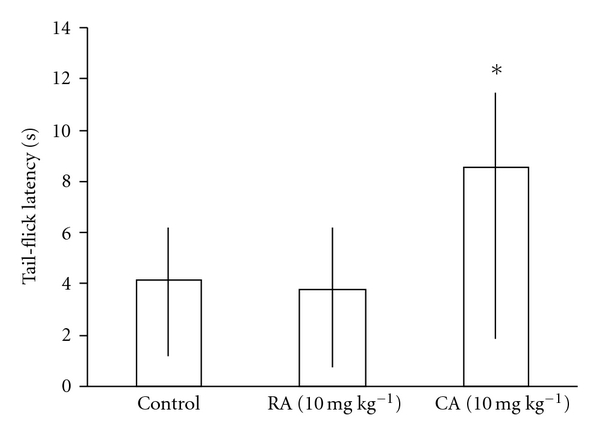
Effect of caffeic acid (CA) and rosmarinic acid (RA) on the tail-flick test in rats. Data expressed as median (interquartiles range) of the tail-flick latencies (*n* = 10 animal/group). Mann-Whitney *U*-test: *P < .05 compared with control group.

Figure 1 shows a significant difference in the group of animals treated with 10 mg/kg caffeic acid in comparison with the other groups. The analysis revelated a high latency to tail-flick characterizing a nociceptive response. There was no difference between control group and animals treated with rosmarinic.

## 4. Discussion

The intrapleural administration of carrageenan into the pleural space leads to pleurisy, an inflammatory process characterized by an immediate recruitment of polymorphonuclear cells (PMN). Carrageenan is a high-molecular-weight sulphated polysaccharide, capable of inducing the release of mediators involved in vascular changes associated with acute inflammation [30]. The inflammation in the respiratory pathway induces histamine, thromboxane A2, leukotrienes, cytokines, and nitric oxide release [31]. 

Rosmarinic acid is a natural phenolic compound contained in many Lamiaceae herbs. The medicinal value of this substance has been well recognized, especially in regard to its antioxidant and anti-inflammatory process. Sabongi et al. [20] observed that rosmarinic acid inhibited pathophysiological changes such as neutrophilic inflammation and edema in the lung, in mice treated with administration of rosmarinic acid (2 mg/body for 3 days). These results suggested that rosmarinic acid inhibits diesel exhaust particles (DEPs) induced lung injury by the reduction of proinflammatory molecule expression. These effects were associated with the changes in proinflammatory cytokine and chemokine expression, which play a crucial role in the initiation and progression of the inflammatory response. Chemokines are a superfamily of small, structurally related chemotactic cytokines involved in leukocyte trafficking and activation [32].

Others studies on its anti-inflammatory activities led to its discovery as an inhibitor of complement [33]. It has been shown to inhibit both the classical and the alternative pathways of complement activation [34, 35] and complement dependent stimulation of prostacyclin synthesis [36]. *In vivo *it is known to inhibit cobra venom factor-induced paw edema [33, 34]. 

Caffeic acid (3,4-dihydroxycinnamic acid) is one of the natural phenolic compounds widely distributed in plant materials such as vegetables, fruits, coffee, and tea [36]. This substance as an antioxidant can scavenge a number of reactive species, including 1,1-diphenyl-2-picryl-hydrazyl free radical (DPPH) [25, 26], peroxyl [23], and hydroxyl radicals [24] as well as superoxide anion, peroxynitrite, and mutagenic compounds such as nitrosamines [24, 26]. Caffeic acid also inhibits 5-lipoxygenase (5-LOX) activity [21], and inhibits protein kinase C (PKC), PKA and nuclear factor-*κ*B (NF-*κ*B) activation induced by ceramides in U937 cells [22]. Others studies have been also reported to have antitumor activity [37, 38], anti-inflammatory properties [39], and anti-HIV replication activity [40]. 

In the present study, we confirmed the anti-inflammatory ability of caffeic and rosmarinic acids in animal model. Experimental evidences obtained in the present study indicated that caffeic acid and rosmarinic acid were able to reduce polymorphonuclear and mononuclear cells migration into pleural space, 4 hours after pleurisy induction by carrageenan. We also observed that animals treated with 10 mg/kg caffeic acid produced an analgesic effect 30 minutes after drug administration on tail-flick model. Our results are in agreement with those reported by Shin et al. 2004 [41] where animals treated with caffeic acid at some dose, significantly increased the latency of the jumping response, and the animals were not able to detect pain by hot plate test. 

Other studies investigating the pharmacological profile of these compounds showed that rosmarinic acid may present an anxiolytic-like activity when used in low doses, without affecting the locomotion, exploration, and motivation [42]. In this work an intermediary doses were chosen based on the literature [42]. The fact that caffeic acid produced analgesic effect in the nociceptive model is indicative that it had both central and periferic antinociception and the mechanism of action could be partially related to lipoxygenase and/or cyclooxygenase of the arachidonic acid cascade and/or opioid receptors [43, 44]. Analgesic effect reflected in tail-flick tests is dependent on centrally action opioid-like analgesics [44, 45]. Taken together, our results obtained in the tail-flick model corroborated that caffeic acid had central analgesic activity, possibly by crossing the blood-brain barrier. 

In our study, we observed the anti-inflammatory and antinociceptive effect was more pronounced in animals treated with caffeic acid as compared to those treated with rosmarinic acid. Germanò et al. [46] studied rats treated with nonhydrolyzed extract of *Trichilia emetica *Vahl and they were detected in plasma levels of caffeic acid at 15 and 30 minutes after, and they suggested its greater absorption *in vivo* by intestinal cells. Baba et al. [47] studying rosmarinic acid bioavailability in orally treated rats, verified maximal serum concentration of this substance 1 hour after administration. Rosmarinic acid was immediately metabolized, and one of its metabolites is caffeic acid. Therefore, we suggest that the anti-inflammatory effect observed in rats treated with rosmarinic acid, is probably due to its breakdown products, such as caffeic acid, as the highest inhibition of leukocyte migration to the primary site of inflammation was obtained with caffeic acid at 10 mg/kg.

 In conclusion, the results of the present study provide further evidences of the anti-inflammatory and analgesic properties of these phenolics compounds, which therefore have potential applications in pain and inflammatory diseases, with caffeic acid presenting the most potent anti-inflammatory and antinociceptive effects in these animal models. However, further studies are needed to establish the mechanism of the observed actions.

## Figures and Tables

**Table 1:** Effect of rosmarinic acid (RA) and caffeic acid (CA) on leukocytes of peripheral blood before and after inflammatory process induction.

Treatment	Total leukocyte (before)	Total leukocyte (after)	Neutrophils (before)	Neutrophils (after)	Lymphocytes (before)	Lymphocytes (after)
Control *n* = 7	7350 ± 3127.9	11175 ± 1607.4*	1183.75 ± 519.5	4779.83 ± 1664.9**	5826.75 ± 2512	5401.3 ± 917.73
RA (5 mg/kg) *n* = 7	7875 ± 2332.76	12850 ± 2202.95**	1743.25 ± 833.16	6013.6 ± 1548.85**	5818.75 ± 1960	6054.42 ± 1816.62
RA (10 mg/kg) *n* = 7	8800 ± 1945.5	14808.33 ± 1120.45^∗∗A^	2057.42 ± 769.85^B^	7007.17 ± 994.97^∗∗B^	6274.42 ± 2267.66	7270.25 ± 1297.25^B^
CA (5 mg/kg) *n* = 7	9100 ± 1801.4	14075 ± 1553.57^∗∗A^	1530.56 ± 1023.2	6659.81 ± 2044.2**	6890.5 ± 1582.5	6307.63 ± 1478
CA (10 mg/kg) *n* = 7	7625 ± 1906.24	12658.33 ± 3598.95^∗∗A^	820.58 ± 387.23	4758.25 ± 2337.54**	6479.3 ± 1746.42	7387.58 ± 1615.63

Mean ± standard error (SEM). Students *t*-test: Compared with control group  ^A^ *P* < .05, ^B^ *P* < .01. Compared between before and after inflammatory induction process *P < .05, **P < .01.

**Table 2:** Migrated leucocytes into pleural exudate in rats treated with caffeic acid (CA) and rosmarinic acid (RA).

Treatment	Total cells	PMN	MN
	×10^6^ cells		
Control *n* = 6	1.56 ± 0.64	1.12 ± 0.57	0.44 ± 0.22
RA (5 mg kg^−1^) *n* = 6	0.34 ± 0.35^A^	0.18 ± 0.14^A^	0.16 ± 0.13^B^
RA (10 mg kg^−1^) *n* = 6	0.30 ± 0.15^A^	0.18 ± 0.10^A^	0.12 ± 0.08^A^
CA (5 mg kg^−1^) *n* = 8	0.53 ± 0.17^A^	0.25 ± 0.12^A^	0.28 ± 0.18
CA (10 mg kg^−1^) *n* = 6	0.11 ± 0.008^A1^	0.005 ± 0.003^A1^	0.006 ± 0.003^A1^

Values are Mean ± S E M. PMN: polymorphonuclear cells. MN: mononuclear cells.

Student's *t*-test: Compared with control group: ^A^ *P* < .05, ^B^ *P* < .01.

ANOVA/Tukey: Compared between groups: ^1^ *P* < .05.
